# Effect of Hospital Teaching Status on Outcomes of Patients With Acute Pancreatitis

**DOI:** 10.7759/cureus.32263

**Published:** 2022-12-06

**Authors:** Hunza Chaudhry, Aalam Sohal, Armaan Dhaliwal, Gagan Gupta, Piyush Singla, Raghav Sharma, Isha Kohli, Dino Dukovic, Jaya Krishna Chintanaboina

**Affiliations:** 1 Internal Medicine, University of California San Francisco, Fresno, Fresno, USA; 2 Hepatology, Liver Institute Northwest, Fresno, USA; 3 Internal Medicine, University of Arizona College of Medicine - Tucson (South Campus), Tucson, USA; 4 Internal Medicine, Dayanand Medical College and Hospital, Ludhiana, IND; 5 Psychiatry, Punjab institute of medical sciences, Jalandhar, IND; 6 Public Health Sciences, Icahn School of Medicine at Mount Sinai, New York, USA; 7 Internal Medicine, Ross University School of Medicine, Bridgetown, BRB; 8 Gastroenterology and Hepatology, University of California San Francisco, Fresno, Fresno, USA

**Keywords:** differences, mortality, teaching status, pancreatitis, national inpatient sample

## Abstract

Introduction

Multiple studies have shown that outcomes of various diseases differ by the hospital teaching status. However, not much is known about the effects of hospital teaching status on outcomes of acute pancreatitis (AP). The aim of this study was to identify if there was an effect of hospital teaching status on the outcomes of AP.

Methods

The National Inpatient Sample (NIS) database was used to identify patients with a discharge diagnosis of AP from 2016 to 2019. Patients were classified according to whether they were admitted to teaching hospitals (TH) or non-teaching hospitals (NTH). Study outcomes were the length of stay (LOS), total hospitalization cost and charge, sepsis, shock, acute kidney injury, ICU admission, and mortality.

Results

A total of 1,689,334 patients were included in the study. Of these, 65.06% were in the TH group, while 34.94% were in the NTH group. Patients admitted to TH had a higher incidence of AKI (18.84% vs. 15.79%, p<0.001), shock (4.32% vs. 2.7%, p<0.001), sepsis (4.48% vs. 3.65%, p<0.001), and ICU admissions (4.78% vs. 2.90%, p<0.001) than NTH. Patients admitted to TH also had a higher length of stay (5.82 vs. 4.54 days, p<0.001) and higher hospitalization charges ($47,390 vs. $65,380, p<0.001). The mortality rate in TH was 2.25% compared to 1.5% in NTH (p<0.001).

Conclusion

Patients admitted to TH had higher mortality as compared to NTH. While the exact reason for this is unknown, it can be partially explained by a higher incidence of AKI, shock, and sepsis. Furthermore, ICU admissions were higher in TH, indicating higher resource utilization.

## Introduction

The US healthcare system can be broadly divided into two major hospital systems, teaching hospitals (TH) and non-teaching hospitals (NTH). According to Health Care Utilization Project (HCUP), the largest collection of longitudinal hospital care data, a hospital is classified as a TH if it has one or more Accreditation Council for Graduate Medical Education (ACGME) approved residency programs or is a member of the Council of Teaching Hospitals (COTH) or has a ratio of full-time equivalent interns and residents to beds of 0.25 or higher [1]. TH have served as a pillar for advancing medicine by promoting research, educating trainees, and delivering care to the underserved population [2]. 

Acute pancreatitis (AP) is inflammation of the pancreas. There are 275,000 admissions for AP yearly, accounting for $2.5 billion US dollars in healthcare expenditure. It is the most common gastrointestinal condition requiring hospitalization [3]. Most cases of AP are benign with a self-limited course. About 10-20% of the patients progress to severe pancreatitis, which is associated with complications and increased mortality risk [4]. AP-related hospitalizations continue to increase, but a recent retrospective analysis showed reduced mortality rates and lower hospitalization costs [5]. Nonetheless, the overall mortality rate can approach 5%, with severe cases having a mortality rate of 30% [6-8].

Prior studies have evaluated the effect of hospital teaching status on outcomes in various disease processes [9-10]. Based on our literature review, no studies have assessed the impact of the teaching status of the hospital on the outcomes of hospitalizations for AP. In this study, we investigated the effect of hospital teaching status on AP outcomes.

## Materials and methods

Data source

The National Inpatient Sample (NIS) database is maintained by the Agency for Healthcare Research and Quality (AHRQ). It is part of the healthcare utilization project (HCUP), which is a family of databases, software tools, and related products developed through a Federal-State-Industry partnership and sponsored by AHRQ. HCUP databases are derived from administrative data and contain encounter-level, clinical and non-clinical information, including all-listed diagnoses and procedures, discharge status, patient demographics, and charges for all patients, regardless of payer (e.g., Medicare, Medicaid, private insurance, uninsured), beginning in 1988. These databases enable research on a broad range of health policy issues, including cost and quality of health services, medical practice patterns, access to health care programs, and outcomes of treatments at the national, state, and local market levels. NIS is the largest inpatient database in the United States and contains data from 20% of all hospitalizations, representing approximately 8 million (unweighted) and 40 million (weighted) hospitalizations yearly. The NIS database contains information regarding clinical data and resource utilization in hospitalized patients while protecting patients' privacy. It includes one primary diagnosis, up to 40 secondary diagnoses, population baseline characteristics, patient comorbidities, and total charges.

Study population

**Figure 1:**
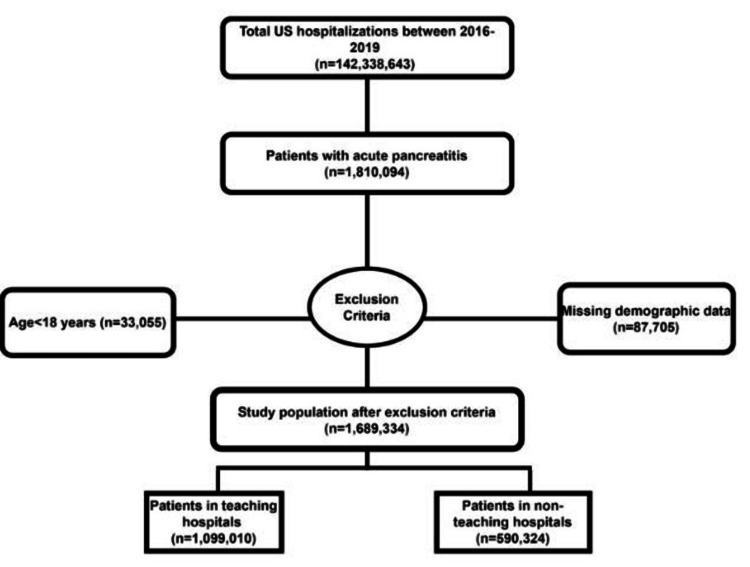
Inclusion flow diagram for the study

We queried the NIS database from 2016 to 2019 using the International Classification of Diseases, Tenth Revision Clinical Modification (ICD-10 CM), for patients with a discharge diagnosis of AP. The ICD-10 codes used in the study are shown in Appendix 1. The hospital's teaching status was ascertained based on prespecified data by HCUP. Patients were classified according to admissions to TH or NTH. Patients who were under 18 or missing information on demographics were excluded from the analysis. This information is presented in Figure 1.

Study outcomes and variables

The categorical outcome measures were sepsis, shock, acute kidney injury (AKI), intensive care unit (ICU) admission, and inpatient mortality. We also measured the difference between continuous variables such as length of stay and total hospitalization charges. Hospital charges are defined as the amount charged by the hospital, before negotiating discounts with insurance companies.

Statistical analysis

Data for continuous variables are presented as population-weighted mean ± SE (standard error), while categorical variables are presented as a total number of patients with percentages. Univariate analysis was performed to assess differences between subjects admitted at TH and NTH. Continuous variables were compared using t-tests, and categorical variables were compared using chi-square tests. We also collected data on Charlson's Comorbidities. This is a well-validated index, which has been used in large administrative data to predict mortality and hospital resource utilization. This index has 17 comorbidities [11].

Hospital-level discharge weights provided by NIS were used to generate national estimates. Categorical variables were compared using the chi-square test, whereas an independent sample t-test was used for continuous variables. Univariate analysis was performed to study the effect of patient demographics, hospital characteristics, Charlson Comorbidities, and etiology of pancreatitis on categorical outcomes. A p-value of 0.1 was considered a cut-off. A multivariate regression model was then built by including all variables found to be significant by univariate analysis to calculate the adjusted odds ratio. Logistic regression was used for categorical outcomes, and linear regression was used for continuous outcomes. A type I error of < 0.05 was considered statistically significant. Data analysis was performed using Stata (StataCorp. 2021. Stata Statistical Software: Release 17).

## Results

Demographics and hospital characteristics

A total of 1,689,334 patients were included in the study. Of these, 65.06% were in the TH group, while 34.94% were in the NTH group. Information on the total number of patients in the study population is presented in Table 1.

**Table 1:** Demographic and Hospital Characteristics of the Study Population

Variable	n (%)
Total number of patients	1,689,334
Mean age in years	53.4 (+/-0.4)
Age	
18-45	547,425 (32.4%)
45-65	681,415 (40.34%)
>65	460,495 (27.26%)
Sex	
Male	879,255 (52.05%)
Female	810,080 (47.95%)
Race	
White	1,082,429 (64.07%)
African American	269,635 (15.96%)
Hispanic	230,690 (13.66%)
Asian/Pacific islander	41,570 (2.46%)
Native American	14,535 (0.86%)
Other	50,475 (2.99%)
Insurance	
Medicare	577,205 (34.17%)
Medicaid	397,725 (23.54%)
Private	509,595 (30.17%)
Self-pay	143,225 (8.48%)
Income	
Lowest quartile	546,560 (32.35%)
Second quartile	448,120 (26.53%)
Third quartile	396,320 (23.46%)
Highest quartile	298,335 (17.66%)
Region	
Northeast	280,530 (16.61%)
Midwest	360,935 (21.37%)
South	691,775 (40.95%)
West	356,094 (21.08%)
Hospital location	
Rural	168,530 (9.98%)
Urban	1,520,805 (90.02%)
Hospital bed size	
Small	385,684 (22.83%)
Medium	513,700 (30.41%)
Large	789,950 (46.76%)

Patients admitted to TH were younger than patients at NTH. Thirty-three percent of the patients in TH were between 18-45 years old as compared to 31.23% in NTH. About 26.6% of the patients in the TH group were >65 years old compared to 28.49% in the NTH group. Racial differences were noted between TH and NTH. Whites comprised 70.23% of the population at NTH compared to 60.77% at TH. TH had a higher proportion of African Americans (17.98% vs. 12.21%) and Hispanics (14.52% vs. 12.04%) than NTH. Patient demographics and hospital characteristics are presented in Table 2.

**Table 2:** Demographics and hospital characteristics of the study population stratified by hospital teaching status

	Non-Teaching Hospitals n (%)	Teaching Hospitals n (%)	p-value
Total number of patients	590,324	1,099,010	
Mean age in years	54.02 (+/-0.06)	53.11 (+/-0.05)	<0.001
Age			<0.001
18-45	184,330 (31.23%)	363,095 (33.04%)	
45-65	237,805 (40.28%)	443,610 (40.36%)	
>65	168,190 (28.49%)	292,305 (26.6%)	
Sex			0.868
Male	307,125 (52.03%)	572,130 (52.06%)	
Female	283,200 (47.97%)	526,880 (47.94%)	
Race			<0.001
White	414,600 (70.23%)	667,830 (60.77%)	
African American	72,080 (12.21%)	197,555 (17.98%)	
Hispanic	71,070 (12.04%)	159,620 (14.52%)	
Asian/Pacific islander	11,960 (2.03%)	29,610 (2.69%)	
Native American	5,915 (1%)	8,620 (0.78%)	
Other	14,700 (2.49%)	35,775 (3.26%)	
Insurance			<0.001
Medicare	211,280 (35.79%)	365,925 (33.3%)	
Medicaid	127,845 (21.66%)	269,880 (24.56%)	
Private	173,995 (29.47%)	335,600 (30.54%)	
Self-pay	55,115 (9.34%)	88,110 (8.02%)	
Income			<0.001
Lowest quartile	204,295 (34.61%)	342,265 (31.14%)	
Second quartile	172,745 (29.26%)	275,375 (25.06%)	
Third quartile	126,865 (21.49%)	269,455 (24.52%)	
Highest quartile	86,420 (14.64%)	211,915 (19.28%)	
Region			<0.001
Northeast	62,715 (10.62%)	217,815 (19.82%)	
Midwest	122,090 (20.68%)	238,845 (21.73%)	
South	272,550 (46.17%)	419,225 (38.15%)	
West	132,970 (22.52%)	223,125 (20.3%)	
Hospital location			<0.001
Rural	168,530 (28.55%)	0	
Urban	421,795 (71.45%)	1,099,010 (1%)	
Hospital bed size			<0.001
Small	115,299 (19.53%)	270,385 (24.6%)	
Medium	179,055 (30.33%)	334,645 (30.45%)	
Large	295,970 (50.14%)	493,980 (44.95%)	
Charlson Comorbidity Index			<0.001
0-1	390,345 (66.12)	682,460 (62.1)	
2	84,160 (14.26)	160,830 (14.63)	
3 or more	115,820 (19.62)	255,720 (23.27)	

Etiology of pancreatitis and comorbidities

Biliary pancreatitis accounted for 20.73% of admissions in TH as compared to 18.18% in NTH. A similar proportion of patients with alcohol-related pancreatitis were admitted between TH and NTH, 21.04% and 21.16%, respectively. A higher proportion of patients with congestive heart failure (9.33% vs. 8.29%), renal disease (12.47% vs. 11.33%), and malignancy (3.7% vs. 2.44%) were admitted to TH compared to NTH. A complete list of etiology and patient comorbidities is presented in Table 3. 

**Table 3:** Etiology and comorbidities of pancreatitis stratified by hospital teaching status COPD- Chronic obstructive pulmonary disease AIDS- Acquired immunodeficiency syndrome

	Non-Teaching Hospitals n (%)	Teaching Hospitals n (%)	p-value
Etiology			
Biliary pancreatitis	107,310 (18.18%)	227,875 (20.73%)	<0.001
Alcohol-related pancreatitis	124,205 (21.04%)	232,500 (21.16%)	0.571
Comorbidities			
Acute myocardial infarction	28,745 (4.87)	57,550 (5.24)	0.0001
Congestive heart failure	48,960 (8.29)	102,575 (9.33)	<0.001
Peripheral vascular disease	23,735 (4.02)	50,810 (4.62)	<0.001
Stroke	10,675 (1.80)	23,865 (2.17)	<0.001
Dementia	16,970 (2.88)	26,355 (2.40)	<0.001
COPD	98,670 (16.71)	182,025 (16.56)	0.37
Rheumatoid disease	12,830 (2.17)	25,645 (2.33)	0.004
Peptic ulcer disease (PUD)	14,965 (2.53)	31,285 (2.85)	<0.001
Mild liver disease	95,000 (16.09)	193,500 (17.61)	<0.001
Moderate/Severe liver disease	16,555 (2.8)	40,360 (3.67)	
Uncomplicated Diabetes	124,770 (21.14)	217,195 (19.76)	<0.001
Complicated diabetes	47,035 (7.97)	99,900 (9.09)	<0.001
Hemiplegia/Paraplegia	2,205 (0.37)	5,745 (0.52)	<0.001
Renal disease	66,905 (11.33)	137,025 (12.47)	<0.001
Malignancy	14,375 (2.44)	40,705 (3.7)	<0.001
Metastatic Cancer	8,885 (1.50)	25,600 (2.33)	<0.001
AIDS	1,820 (0.30)	6,005 (0.54)	<0.001

Outcomes

Length of Stay

The length of stay in TH was 5.82 days, while in NTH it was noted to be 4.54 days. Patients admitted to TH had a statistically significant higher length of stay compared to NTH (adjusted coefficient: 0.95, 95% CI-0.88-1.02, p<0.001).

Mean Hospitalization Charge

In patients admitted to NTH, the mean charge was $47,390 as compared to $65,380 in patients at TH, respectively. Patients admitted to TH had significantly higher hospitalization charges than NTH (adjusted coefficient: $10,9827.8, 95% CI- $9,206.00-$12,625.47, p<0.01).

Shock

The total incidence of shock in the study population was 3.76%. The incidence of shock in TH was 4.32% compared to 2.70% in NTH. There was a statistically significant increase in the likelihood of shock in TH (aOR:1.43, 95% CI-1.36-1.50, p<0.001).

Sepsis

The total incidence of sepsis in the study population was 4.19%. The incidence of sepsis in TH was 4.48% compared to 3.65% in NTH. A statistically significant increase was noted in the likelihood of shock at TH compared to NTH (aOR:1.21, 95% CI-1.14-1.28, p<0.001).

ICU Admission

ICU admissions accounted for 4.12% of total admissions. In our study, 4.78% of patients in TH and 2.90% in NTH required ICU admissions. Patients admitted to TH had a statistically significant increase in the likelihood of ICU admissions (aOR:1.45, 95% CI-1.38-1.52, p<0.001).

AKI

A total of 17.78 % of patients had AKI. In our study, 18.84% of the patients in TH and 15.79% in NTH developed AKI. Patients admitted to TH were more likely to develop AKI than NTH (aOR:1.19, 95% CI-1.15-1.22,p<0.001).

All-Cause Mortality

**Figure 2:**
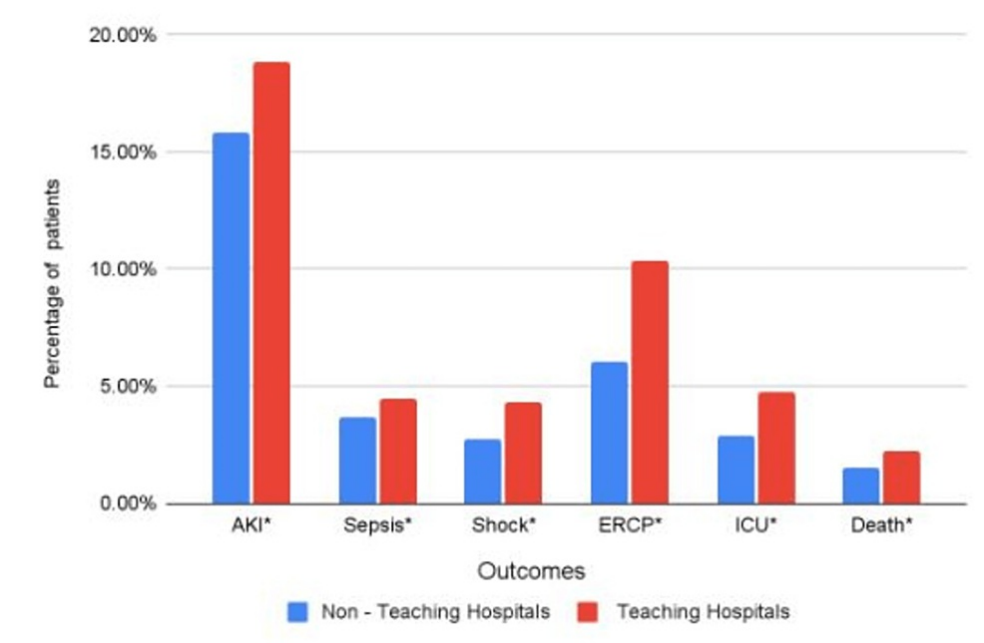
Comparison of categorical outcomes between teaching and non-teaching hospitals. * refers to statistical significance. AKI- Acute Kidney Injury, ERCP - Endoscopic Retrograde Cholangiopancreaticography, ICU- Intensive Care Unit

The total mortality in the study population was 1.99%. The incidence of mortality in TH was 2.25% as compared to 1.5% in NTH. A statistically significantly higher mortality was noted in TH compared to NTHs (aOR:1.37,95% CI-1.27-1.45 p<0.001). The differences in mortality and other categorical outcomes between TH and NTH groups are presented in Figure 2.

## Discussion

In our retrospective study, 1.6 million patients were admitted with AP between 2016 and 2019. Of them, 65.06% were admitted to TH, while 34.94% were admitted to NTH. The study revealed that patients admitted to TH had a higher likelihood of sepsis, AKI, and shock. They also had more comorbidities compared to NTH. Similar to our study, Yiadom et al. revealed a higher median case acuity in patients admitted to TH compared to NTH [12]. THs can also serve as referral centers for a higher level of care due to the availability of subspecialists [13]. There is a possibility that some of the severe pancreatitis cases could have been transferred from NTH to TH. 

Patients admitted to TH had significantly higher hospitalization charges than those admitted to NTH. This could be due to the possibility that patients admitted to TH required a higher level of care. There is also a possibility that more cholecystectomies and ERCP’s were performed at TH as more patients with biliary pancreatitis received treatment at TH than NTH. A recent study by Rotundo et al. revealed that patients who required therapeutic ERCP had a higher length of stay and hospitalization charges in TH than NTH [14]. We believe that higher rates of ERCP in TH might have also led to these differences in hospitalization charges.

Rising admission rates for AP in the last few years have led to increased utilization of healthcare resources. Researchers examining the expenses incurred by patients found that while in-hospital charges were higher at TH, 30-day post-hospitalization charges were lower at TH [15]. This reduction can be credited to improved post-discharge planning and better care processes. Therefore, appropriately allocating limited resources is paramount in managing this rising demand. 

We note the following limitation of this study. NIS lacks objective data such as laboratory tests, thus limiting the ability to calculate the severity of illness scores such as APACHE or BISAP scores. Secondly, information on treatment therapies such as the amount of intravenous fluids administered is not provided in NIS, which is an important confounder and can affect patient outcomes. In addition, NIS does not provide information on readmissions therefore, we can not track readmissions. As a result, it is difficult if it was an initial or recurrent episode. Due to the nature of the database, it is difficult to ascertain if the treatment was led by an expert or trainee, which can impact the outcomes. The major strength of this study is the large study population size from several hospitals across the country, which excludes selection bias based on the demographics. Our findings should be validated in a prospective cohort that captures more granular clinical data. 

## Conclusions

Our retrospective study highlights the differences in the outcomes of acute pancreatitis in TH and NTH based on several factors discussed above. Patients admitted to TH had higher severity, as evidenced by a higher incidence of sepsis, shock, AKI, and ICU admission. Patients admitted to TH had a higher risk of mortality. Furthermore, they also had higher resource utilization, as evidenced by the higher length of stay and total hospitalization charges. Our study should be validated in further prospective studies, and factors contributing to these differences should be examined and reported. 

## Appendices

Appendix 1 

**Table 4:** ICD-10 codes used in the study

Variables	ICD-10 codes
Pancreatitis	K85
Biliary Pancreatitis	K85.1
Alcohol-related Pancreatitis	K85.2
Acute Myocardial Infarction	I21.x, I22.x, I25.2
CHF	I09.9,I11.0, I13.0, I13.2, I25.5, I42.0, I42.5-I42.9, I43.x, I50.x, P29.0
Peripheral Vascular DIsease	I70.x, I71.x, I73.1, I73.8, I73.9, I77.1, I79.0, I79.2, K55.1, K55.8, K55.9, Z95.8, Z95.9
Stroke	G45.x, G46.x, H34.0, I60.x-I69.x
Dementia	F00.x-F03.x, F05.1, G30.x, G31.1
COPD	I27.8, I27.9, J40.x-J47.x, J60.x-J67.x, J68.4, J70.1, J70.3
Rheumatoid Disease	M05.x, M06.x, M31.5, M32.x-M34.x, M35.1, M35.3, M36.0
Peptic Ulcer Disease	K25.x-K28.x
Mild Liver Disease	B18.x, K70.0-K70.3, K70.9, K71.3-K71.5, K71.7, K73.x, K74.x, K76.0, K76.2-K76.4, K76.8, K76.9, Z94.4
Uncomplicated DIabetes	E10.0, E10.l, E10.6, E10.8, E10.9, E11.0, E11.1, E11.6, E11.8, E11.9, E12.0, E12.1, E12.6, E12.8, E12.9, E13.0, E13.1, E13.6, E13.8, E13.9, E14.0, E14.1, E14.6, E14.8, E14.9
Complicated DIabetes	E10.2-E10.5, E10.7, E11.2-E11.5, E11.7, E12.2-E12.5, E12.7, E13.2-E13.5, E13.7, E14.2-E14.5, E14.7
Hemiplegia/Paraplegia	G04.1, G11.4, G80.1, G80.2, G81.x, G82.x, G83.0-G83.4, G83.9
Renal Disease	I12.0, I13.1, N03.2-N03.7, N05.2-N05.7, N18.x, N19.x, N25.0, Z49.0-Z49.2, Z94.0, Z99.2
Malignancy	C00.x-C26.x, C30.x-C34.x, C37.x-C41.x, C43.x, C45.x-C58.x, C60.x-C76.x, C81.x-C85.x, C88.x, C90.x-C97.x
Moderate/Severe Liver Disease	I85.0, I85.9, I86.4, I98.2, K70.4, K71.1, K72.1, K72.9, K76.5, K76.6, K76.7
Metastatic Cancer	C77.x-C80.x
AIDS	B20.x-B22.x, B24.x
Shock	R65.10,R65.11,R65.20
Sepsis	R6521,R571,R578,R579
Pressor	3E030XZ,3E033XZ, 3E040XZ, 3E043XZ, 3E050XZ, 3E053XZ, 3E060XZ,3E063XZ
Mechanical Ventilation	5A1935Z,5A1945Z,5A1955Z
AKI	N17.0, N17.1, N17.2, N17.8, N17.9
ERCP	0FQC7ZZ, 0FQC8ZZ,0F758ZZ,0F768ZZ,0F778ZZ,0F7C8DZ,0F7C8ZZ,0F958ZZ, 0F968ZZ 0F978ZZ, 0F988ZZ,0F998ZZ, 0F9C8ZZ, 0F9D8ZZ,0F9C80Z,0F9980Z, 0F758DZ, 0F768DZ,0F778DZ, 0F788DZ,0F798DZ,0FHB4DZ, 0FHB8DZ, 0FC58ZZ, 0FC68ZZ, 0FC78ZZ, 0FC88ZZ, 0FC98ZZ, 0FCC8ZZ, 0FF48ZZ, 0FF58ZZ, 0FF68ZZ, 0FF78ZZ, 0FF88ZZ, 0FF98ZZ, 0FFC8ZZ, 0F959ZX, 0F968ZX, 0F978ZX, 0F988ZX, 0F998ZX,0F968ZX,0FB48ZX,0FB58ZX,0FB68ZX,0FB78ZX,0FB88ZX,0FB98ZX, 0FBC8ZX, 0FHD8DZ, 0FCD8ZZ, 0F7D8ZZ, 0F758DZ, OF778DZ, 0F798DZ, 0F788DZ, 0F7D8DZ, 0FPB8DZ, 0FPD8DZ
